# Characterization of Microbial Growth, Pathogen Presence, and Histamine Accumulation in Chilled Rainbow Trout and Mackerel Samples Collected from Romanian Markets

**DOI:** 10.3390/pathogens14060580

**Published:** 2025-06-11

**Authors:** Vida Silviu, Alexandra Tabaran, Oana Lucia Crişan Reget, Mihaela Niculina Duma, Luciana Cătălina Panait, Sorin Daniel Dan

**Affiliations:** 1 Animal Breeding and Food Safety Department, Faculty of Veterinary Medicine, University of Agricultural Sciences and Veterinary Medicine, Manastur Street No. 3-5, 400372 Cluj-Napoca, Romania; silviuvida@yahoo.com (V.S.); oana.reget@usamvcluj.ro (O.L.C.R.); luciana.rus@usamvcluj.ro (L.C.P.); sorindan@usamvcluj.ro (S.D.D.); 2 Laboratory of Food Microbiology, Sanitary Veterinary Directorate for Food Safety, 400621 Cluj-Napoca, Romania; duma.mihaelacj@yahoo.com

**Keywords:** microbial spoilage, histamine, rainbow trout, mackerel, chilling storage, microbial characterization, safety

## Abstract

This study aimed to evaluate microbial growth, pathogen presence, and histamine production in rainbow trout and mackerel stored on flaked ice over their shelf life. A total of 72 fish samples (rainbow trout and mackerel) were analyzed across four storage intervals (day 1, 3, 9, 12/11) on flaked ice. TVC increased from 2.59 to 5.04 log cfu/g in rainbow trout and from 3.18 to 4.88 log cfu/g in mackerel over the storage period. Significant increases were observed in *Pseudomonas*, *Aeromonas*, and *Enterobacteriaceae* populations, especially after the ninth day. Microbial identification revealed spoilage-associated bacteria, such as *Pseudomonas fluorescens* and *Aeromonas salmonicida*, as well as opportunistic pathogens, including *Francisella tularensis*, *Yersinia* spp., and *Chromobacterium violaceum*. Histamine levels rose with storage time but remained below toxic thresholds (<200 mg/kg), peaking at 1.56 mg/kg in trout and 1.87 mg/kg in mackerel. A strong positive correlation was found between TVC and histamine levels (Pearson’s r = 0.85 for trout, 0.82 for mackerel). Proper hygiene and storage are crucial, and consumption is recommended before day 9 of storage on flaked ice. Hygiene measures remain essential to minimize contamination risks and preserve product safety.

## 1. Introduction

Despite the challenges confronting the aquaculture industry, including climate change and resistant pathogens, the demand for fish continues to be substantial [1]. Fish is a valuable source of lean protein, omega-3 fatty acids, and essential minerals, making it particularly beneficial for pregnant women, children, and the elderly [2]. Therefore, it is crucial to be aware of its potential to carry pathogenic microorganisms. In Romania, although fish is an integral part of the diet, its consumption is still relatively low compared to the European average, despite the country’s rich aquatic resources [1]. Raising awareness of the health benefits of fish, coupled with improvements in hygiene standards throughout the production process to ensure safety across the entire supply chain, could promote higher consumption in Romania and beyond.

The microbiota present on freshly caught fish is primarily influenced by the microbial content of the water in which the fish are caught or stored, as noted by various researchers [3,4,5]. Pollution in the waters, particularly fecal contamination, can significantly increase the microbial load, with a higher concentration of harmful bacteria such as Enterobacteriaceae being commonly found in polluted waters [6,7].

The microbial composition of fish varies depending on their habitat, with temperate water fish primarily harboring Gram-negative bacteria from genera like Flavobacterium, Pseudomonas, Vibrio, Escherichia, and Aeromonas [8,9,10]. Some Gram-positive bacteria, including Bacillus, Micrococcus, and Staphylococcus, can also be present [11,12]. Marine fish, in particular, are dominated by bacteria from the genera Vibrio, Acinetobacter, and Escherichia [13,14], whereas freshwater fish tend to harbor species like Aeromonas, Pseudomonas, and Vibrio [15,16]. This microbial diversity highlights the potential risks associated with seafood consumption, as these bacteria could have implications for human health, especially in cases where water quality is compromised.

Histamine production in fish is linked to the presence of histamine decarboxylase, an enzyme produced by certain bacteria [17]. Not all bacteria on fish produce this enzyme, and it is used by bacteria to maintain an optimal intracellular pH under acidic conditions [18]. The enzyme exists in two types, depending on whether the bacteria are Gram-positive or Gram-negative, with Gram-negative bacteria producing pyridoxal phosphate-dependent histidine decarboxylase and Gram-positive bacteria producing pyruvoyl-dependent histidine decarboxylase, especially in spoilage-associated bacteria [5]. Various bacteria found on fish, including *Proteus morganii*, *Escherichia coli*, *Vibrio alginolyticus*, *Salmonella* spp., and others, are known to produce histamine decarboxylase, with certain species like *Proteus morganii* and *Escherichia coli* being particularly efficient at producing histamine [17]. This can lead to scombroid poisoning in consumers [17].

In live, healthy fish, bacteria are not present in the muscle tissue, but after death, bacteria proliferate and invade the flesh [19]. The rate of bacterial invasion is influenced by the temperature and the level of bacterial contamination on the fish’s skin. The muscles near the gills are most likely to contain histamine-producing bacteria, which could be transferred from the gills or digestive tract [20]. Contamination can occur at various stages, from fishing and processing to transport and consumer handling. Elevated temperatures during storage and transport, or when fish are kept in nets for extended periods, promote bacterial multiplication and histamine production [21].

Fish, in addition to spoilage microorganisms, can harbor pathogens such as *Listeria monocytogenes*, *Clostridium botulinum*, *Vibrio* spp., *Yersinia* spp., *E. coli*, and *Salmonella* species [22,23,24,25]. These pathogens are typically classified into two groups: those native to aquatic environments and those resulting from water pollution and human activities, including poor hygiene during capture, handling, and transportation [26]. Some of these pathogens, like *Listeria monocytogenes*, *Clostridium botulinum*, and *Vibrio vulnificus*, are associated with serious, potentially fatal diseases. *Listeria monocytogenes* and *Yersinia* spp. pose a greater risk due to their ability to survive outside a host for extended periods [23]. The absence of heat treatment in most fish products before consumption further increases the risk of foodborne outbreaks. Good hygiene practices (GHPs) are crucial to ensuring the safety of fish and fish products [21].

The aim of the study was to conduct a microbial risk assessment for contamination in rainbow trout (*Oncorhynchus mykiss*) and mackerel (*Scomber scombrus*) stored on flaked ice, focusing on spoilage microorganisms and pathogens, as well as histamine production. The study objectives included determining total viable counts, *Enterobacteriaceae*, *Pseudomonas*, and *Aeromonas* counts; isolating bacterial populations; detecting antimicrobial and virulence genes in pathogens; and measuring histamine levels using the HPLC method.

## 2. Materials and Methods

### 2.1. Sample Collection

The experiment was conducted on whole fish stored on flaked ice throughout their shelf life, at an average temperature of −0.5 °C. Between March and April 2024, 36 trout and 36 mackerel samples were purchased from a specialized retailer. The average size of trout was 350 g with an average length of 30 cm, while for mackerel, the average size was 300 g, and the average length was also 30 cm. For rainbow trout, four storage durations were selected, ranging from one to twelve days on flaked ice, at three-day intervals. For mackerel, storage times ranged from one to eleven days under the same conditions and intervals. As a result, fish were analyzed on the first, third, ninth, and final day of shelf life on flaked ice. For each storage time, three individual samples of mackerel and three of rainbow trout were analyzed. In addition, 12 rainbow trout samples were stored at −18 °C for 6 months in order to assess the microbial load and histamine production for this category of food. For each storage time, the three fish were sampled for the microbiological analysis and were then frozen at −18 °C before being sent to the laboratory in order to determine the histamine level. The entire experiment was replicated three times (12 samples/replicate).

### 2.2. Total Viable Count

A tenfold dilution series was made in the range of 10^−1^ to 10^−6^ for each sample. Test tubes were filled with 9 mL of buffered peptone 0.1% and 0.85% sodium chloride. Plate count agar (PCA) agar (Oxoid, UK) was used for the enumeration of the total viable count. Using sterile pipettes, 1.0 mL from every serial dilution was aseptically transferred onto the surface of two petri plates, and then 15 mL of molted PCA agar (Oxoid, UK) (47 °C) was poured in the plates and gently homogenized. The plates were incubated for 48–72 h at 30 ± 1 °C. Detection limit in the case of total viable count was 1 log cfu/g.

### 2.3. Pseudomonas/Aeromonas Count

A tenfold dilution series was made in the range of 10^−1^ to 10^−6^ for each sample. Test tubes were filled with 9 mL of buffered peptone 0.1% and 0.85% sodium chloride. Glutamate starch agar (GSP agar) (Merck, Darmstadt, Germany) was used for the enumeration of *Pseudomonas* and the *Aeromonas* count. *Aeromonas* spp. colonies were identified on the glutamate starch agar as yellow colonies, while *Pseudomonas* spp. were identified as red colonies. In the case of the *Pseudomonas*/*Aeromonas* count, 0.1 mL of inoculum was transferred onto the surface of GSP agar (Merck, Darmstadt, Germany). The inoculum was spread rapidly over the entire agar surface using a thin bent glass rod. The glass rod was sterilized by placing it in 96% ethanol or methanol and then flamed until all the alcohol had evaporated. The plates were incubated for 48–72 h at 30 ± 1 °C. Five specific colonies for both *Aeromonas* and *Pseudomonas* were used for the confirmation based on cultural morphology (Gram staining), oxidase, and biochemistry using VITEK^®^ 2 Compact system. Detection limit in the case of total viable count was 1 log cfu/g.

### 2.4. Enumeration of the Enterobacteriaceae

A tenfold dilution series was made in the range of 10^−1^ to 10^−6^ for each sample. Test tubes were filled with 9 mL of buffered peptone 0.1% and 0.85% sodium chloride. Violet red bile glucose agar (VRBG agar) (Oxoid, Basingstoke, UK) was used to isolate and enumerate the *Enterobacteriaceae*. A total of 1 mL of each dilution was aseptically transferred with a sterile pipette to a petri plate, and 15 mL of molten violet red bile glucose agar (VRBG) (Oxoid, Basingstoke, UK) was added. VRBG was kept before use in a water bath at 50 °C. The agar and the inoculum were carefully mixed in a circular fashion and allowed to set for 15 min for solidification. The plate was then incubated at 37 ± 1 °C for 24–26 h. Five specific colonies for Enterobacteriaceae were used for the confirmation based on cultural morphology (Gram staining) and oxidase and glucose test. The detection limit in the case of the Enterobacteriaceae count was 1 log cfu/g.

### 2.5. Classical Isolation Protocol for Potential Pathogen Strains

#### 2.5.1. Isolation of *Salmonella* spp.

The isolation protocol for *Salmonella* spp. followed strictly the steps recommended by the International Organization for Standardization (ISO) 6579 [27]. Briefly, the fish samples (25 g) were previously homogenized in buffered peptone water (225 mL) with a laboratory blender (Stomacher 400, Seward Ltd., Worthing, UK). The incubation was performed for 24 h at 37 °C, followed by inoculation on *Rappaport-Vassiliadis* (RV) green broth (LabM Limited, Heywood, UK) and incubated for 24 h at 42 °C. The second pre-enrichment broth used was selenite cysteine (SC) broth (LabM Limited, Heywood, UK), and the incubation was again at 37 °C for 24 h. From both enrichment broths obtained, 1 mL was streaked onto brilliant green-phenol red-lactose-sucrose (BPLS) agar (Merck, Darmstadt, Germany) and xylose lysine deoxycholate (XLD) agar (Oxoid, Basingstoke, UK). Following the incubation at 37 °C for 24 h, presumptive *Salmonella* colonies were selected for DNA extraction and molecular confirmation. The identification of *Salmonella* spp. was also performed by biochemical testing on the Mini Vidas Automated analyzer (Biomerieux/Craponne, France) (Biomerieux/Craponne, France) according to the manufacturer’s instructions.

#### 2.5.2. Isolation of *Listeria* spp.

The bacterial isolation protocol was performed according to the horizontal detection and counting method for *L. monocytogenes* [28]. Briefly, 25 g of each sample investigated was inoculated in 225 mL of selective supplement half Fraser broth (Sharlau, Sentmenat, Spain) and then incubated for 25 ± 1 h at 30 ± 1 °C. Afterward, a second enrichment was performed, which consisted of adding 0.1 mL of the broth culture in 10 mL of full-strength Fraser broth (UVM II Selective Supplement Scharlau/Spain) and incubation at 37 °C for 24 ± 2 h. A loopful of each of the half- and full-strength Fraser broths was plated on the chromogenic agar ALOA (Scharlau/Spain) and Oxford agar (Merck, Darmstadt, Germany). All the plates were incubated in aerobic conditions at 37 °C for 24–48 h.

The identification of *Listeria monocytogenes* was also performed by biochemical testing on the Mini Vidas Automated analyzer (Biomerieux/Craponne, France) according to the manufacturer’s instructions.

### 2.6. Microbial Population Isolation Using the VITEK^®^ 2 Compact System

This automatic method was used to analyze the microbial population of the fish samples examined, and the protocol followed specifically the instructions of the manufacturer. Briefly, from each specific colony developed on PCA, GSP, and VRBG media, a suspension of microorganisms was prepared, and then brought to a convenient density, which differs depending on the bacteria we want to identify. The sterile plastic tubes were placed in a box equal to the number of samples to be identified, in which sterile saline was added. With the help of a sterile pipette, a small amount of bacterial culture was collected, which was homogenized in the saline test tube. The turbidity was then checked using the DensiCheck turbidimeter (Biomerieux/Craponne, France). After preparation of the suspension, the cards are inoculated with the suspension of microorganisms using a vacuum apparatus. Each sterile tube containing the suspension of microorganisms is placed in the box, and in the neighboring slot, the identification card is placed by inserting the transfer tube into the prepared suspension. Both Gram-negative bacteria and Gram-positive cards were used for bacterial identification.

### 2.7. Mini Vidas Automated Analyzer for the Detection of Pathogenic Strains

This procedure applies to the detection of microbiological contamination with bacteria of the pathogens in products intended for human consumption through the ELFA (enzyme-linked fluorescent assay) technique using the Mini Vidas automated system (Biomerieux/Craponne, France). The VIDAS^®^ *Salmonella* (SLM) automatic EIA test for the detection of *Salmonella* and *Listeria* in food and environmental samples employs a mix of highly specific capture antibodies against both O and H antigens. This allows for the detection of both motile and non-motile *Salmonella* species. Sample preparation—*Salmonella* detection: Transfer 500 µL of the M broth into the well of the VIDAS SLM reagent strip. Keep the remaining broth (MKTT and RVS) at 2–8 °C to confirm positive results if needed. Listeria detection: Transfer 500 µL of Fraser broth into the well of the VIDAS LMO2 reagent strip. Once the sample is placed in the VIDAS system, the process is automatically controlled by the computer. Fluorescence is measured for each sample twice in the reading cuvette of the reagent strip. Baseline reading of the substrate cuvette occurs before the introduction of the cone into the substrate. Measurement occurs after the substrate has incubated with the enzyme remaining inside the cone. Result calculation: The relative fluorescence value (RFV) is calculated by subtracting the baseline reading from the final reading. Results interpretation: The outcome is reported as either positive or negative for the tested sample. Each sample is analyzed based on 25 g (or 25 mL) of the product under examination.

### 2.8. Histamine Determination by High Performance Liquid Chromatography

Each sample was frozen and then sent by transporter to the National Sanitary Veterinary and Food Safety Laboratory in Tulcea County (Romania) for analysis. The determination of histamine was performed using the reference method [29]. Briefly, an aqueous solution is made from homogenized solid samples. Both the suspended samples and the liquid samples are purified by treatment with perchloric acid. After filtration and dilution with water, the extracts are analyzed by liquid chromatography on a column to determine biogenic amines, having as the mobile phase a phosphate buffer solution with pH = 6.00. Histamine (and other primary amines) is determined quantitatively by post-column derivatization with o-Phthalaldehyde (OPA). Peaks separated by histamine are measured by fluorescence (excitation at 330 nm and emission at 465 nm). From each fish, 10 g of sample was taken with a precision of 0.001 g, located 3 cm behind the gills as it was shown to be a site of predilection for histamine-producing bacteria [30,31]. The entire experiment was replicated three times. The HPLC system (Tosoh, Liege, Belgium) used an ALKION cation exchange column, an ALKION pre-column, and a post-derivatization system. A mobile phase chromatogram was performed for 22 min. The picks of histamine were recorded and then quantified by integration. Calculation of the results was performed using the formula histamine (mg/kg) = (HistC × 100)/m, where CHist = histamine concentration (mg/kg) of the injected solution and m = mass of the sample taken (10 g in our study).

### 2.9. PCR Confirmation of Pathogen Strains

The DNA extraction was performed directly from specific colonies developed on VRBG agar (for pathogenic *E. coli* detection), XLD agar (for pathogenic *Salmonella* detection), and ALOA agar (for *Listeria monocytogenes* detection). The DNA extraction protocol followed strictly the steps described by Mihaiu et al., 2014 [32].

For *Salmonella* spp. confirmation, the identification of *ompC* was performed in a final volume of 25 μL containing 25 pmol of each primer, 12.5 μLof MasterMix (Bioline, London, UK), 4 μL of DNA template, and 6.5 μL PCR water grade (Sigma, Saint Louis, MO, USA). The PCR conditions were initial denaturation at 95 °C for 4 min, followed by 35 cycles of denaturation at 95 °C for 30 s, annealing at 58 °C for 30 s, and 1 min at 72 °C, and final elongation at 72 °C for 5 min. A PCR multiplex protocol was used also for identification of possible *Salmonella* Typhimurium and *Salmonella* Enteritidis. The primer sequences used and PCR amplification protocol were previously described by Mihaiu et al., 2014 [32]. For the positive control, *Salmonella* Typhimurium ATCC 14028 and *Salmonella* Enteritidis ATCC 13076 strains were used.

For *Listeria monocytogenes* identification, we followed the steps previously described by Duma et al., 2024 [33]. Briefly, the 25 µL PCR reaction consisted of 2.5 µL bacterial DNA, 12.5 µL of 2× QIAGEN Multiplex PCR Master Mix (Qiagen, Hilden, Germany), and 10 µL of primers mix formed by 0.4 µM (forward and reverse primers) for lmo1118, 0.4 µM for lmo0737, orf 2819 and orf 2110, 0.1 µM for prs and 0.2 µM for prf A. The multiplex PCR comprised a step for pre-denaturation and polymerase activation at 95 °C for 5 min, a step of 40 amplification cycles (denaturation at 95 °C for 20 s, hybridization at 54 °C for 40 s, and elongation at 72 °C for 90 s) followed by a final step at 72 °C for 7 min for elongation. The electrophoresis gel was prepared from 2% agarose (Bioline, London, UK) in TAE (Bioline, UK). Electrophoresis was performed for 1 h and 30 min at 100 V, using 10 µL of DNA ladder (Promega, Southampton, UK) and 10 µL of amplicons, all of them mixed with 2 µL of 6× dual-action nontoxic fluorescent nucleic acid stain and loading dye (RedSafe, Bioline, UK).

### 2.10. Statistical Analysis

The results were analyzed statistically using the Origin 8.5 program, using the ANOVA single-factor categorical analysis system. The mean (X) value and standard deviation (SD) were calculated individually for total viable count parameters and histamine level. A linear regression fit (TVC versus histamine) was also plotted, supported by statistical indicators including Pearson’s correlation coefficient (r), R^2^, and adjusted R^2^ values. The level of significance used in this research was *p* < 0.05.

## 3. Results

### 3.1. Results Total Viable Counts

**Figure 1:**
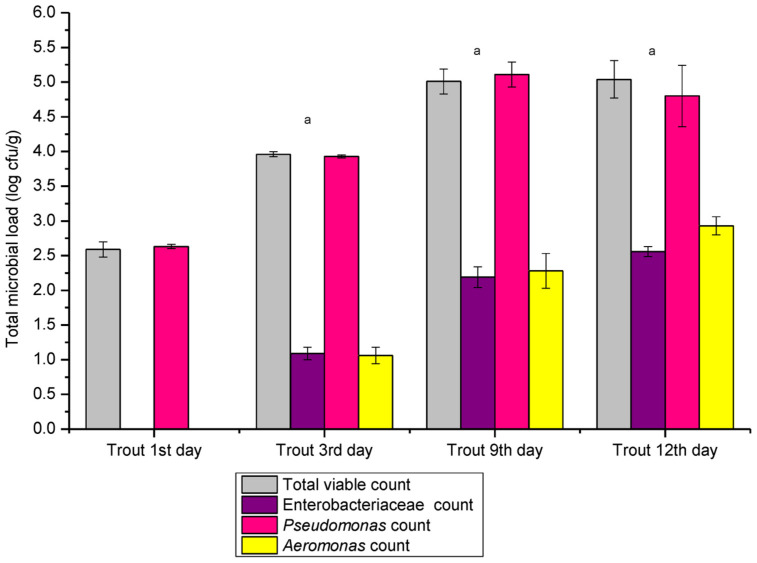
Total viable count, *Enterobacteriaceae*, *Pseudomonas*, and *Aeromonas* count in the case of rainbow trout on flaked ice (*Oncorhynchus mykiss*) during the shelf life (n = 36, 12 samples/replicate). ^a^ Significant differences (*p* < 0.05) when 1st day compared with the 3rd, 9th, and 12th day.

The microbial load of rainbow trout stored on flaked ice throughout its shelf life is shown in Figure 1. The total viable count increased from 2.59 ± 0.11 log cfu/g on day 1 to 5.04 ± 0.27 log cfu/g by day 12. Significant differences were observed between the total viable count on day 1 and day 3 (*p* = 0.00016), and between day 3 and day 9 (*p* = 0.0189), but no significant difference was found between the counts on day 9 and day 12. The *Enterobacteriaceae* count also rose over the shelf life, from below 1.0 log cfu/g on day 1 to 2.56 ± 0.072 log cfu/g on day 12. Significant differences were noticed between the counts on days 3 and 9 (*p* = 0.0092), and on days 9 and 12 (*p* = 0.011). The *Pseudomonas* count increased from 2.63 ± 0.03 log cfu/g on day 1 to a peak of 5.11 ± 0.18 log cfu/g on day 9. Significant differences were found between days 1 and 3 (*p* = 0.000001) and between days 3 and 9 (*p* = 0.012), but no significant differences were observed between days 9 and 12 (*p* = 0.3). The *Aeromonas* count increased from below 1.0 log cfu/g on day 1 to 2.93 ± 0.13 log cfu/g on day 12. Significant differences were noted between days 3 and 9 (*p* = 0.003), but no significant differences were observed between days 9 and 12 (*p* = 0.15). For frozen trout samples (n = 12), the total viable count was 3.36 ± 0.037 log cfu/g, the *Pseudomonas* count was 3.34 ± 0.04 log cfu/g, and the *Aeromonas* count was 1.99 ± 0.044 log cfu/g. No *Enterobacteriaceae* were detected in the samples.

**Figure 2:**
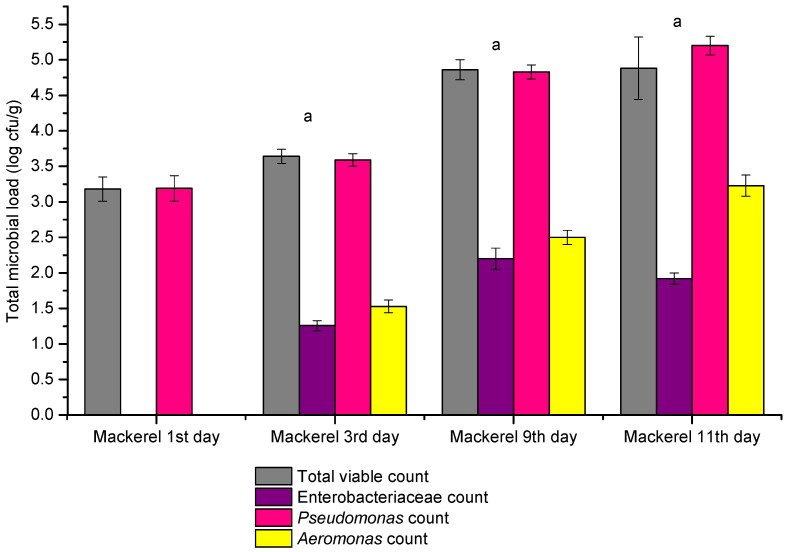
Total viable count, *Enterobacteriaceae*, *Pseudomonas*, and *Aeromonas* count in the case of mackerel on flaked ice (*Scomber scombrus*) during the shelf life (n = 36, 12 samples/replicate). ^a^ Significant differences (*p* < 0.05) when 1st day compared with the 3rd, 9th, and 11th day.

The results regarding the microbial load of the mackerel on flaked ice during its shelf life are depicted in Figure 2. Total viable count increases during the shelf life, from 3.18 ± 0.17 log cfu/g on the first day to 4.88 ± 0.44 log cfu/g on the eleventh day. Significant differences were recorded between the first day and the third day (*p* = 0.016) and between the third and the ninth day (*p* = 0.0088), but no difference was noted between the ninth and the eleventh day (*p* = 0.587). The *Enterobacteriacea* count increases during the shelf life, from below 1.0 log cfu/g on the first day to a maximum of 2.2 ± 0.15 log cfu/g on the ninth day. Significant differences were recorded between the third and ninth day (*p* = 0.0103), but no significant differences were noted between the ninth and eleventh day (*p* = 0.072). The *Pseudomonas* count increases during the shelf life, from 3.19 ± 0.18 log cfu/g on the first day to 5.2 ± 0.13 log cfu/g on the twelfth day. Significant differences were recorded between the first and third day (*p* = 0.022), between the third and ninth day (*p* = 0.002), and between the ninth and eleventh day (*p* = 0.034). The *Aeromonas* count increases during the shelf life, from below 1.0 log cfu/g on the first day to 3.23 ± 0 to 15 log cfu/g on the twelfth day. Significant differences were noted between the first and third day (*p* = 0.0029), between the third and ninth day (*p* = 0.0026), and between the ninth and eleventh day (*p* = 0.017). Regarding sensorial changes, at the end of the experiment, both species (trout and mackerel) exhibited a thin layer of slime on the skin surface and a reddish-greyish discoloration of the gills; however, no off-odor was detected.

### 3.2. Microbial Diversity Characterisation

**Figure 3:**
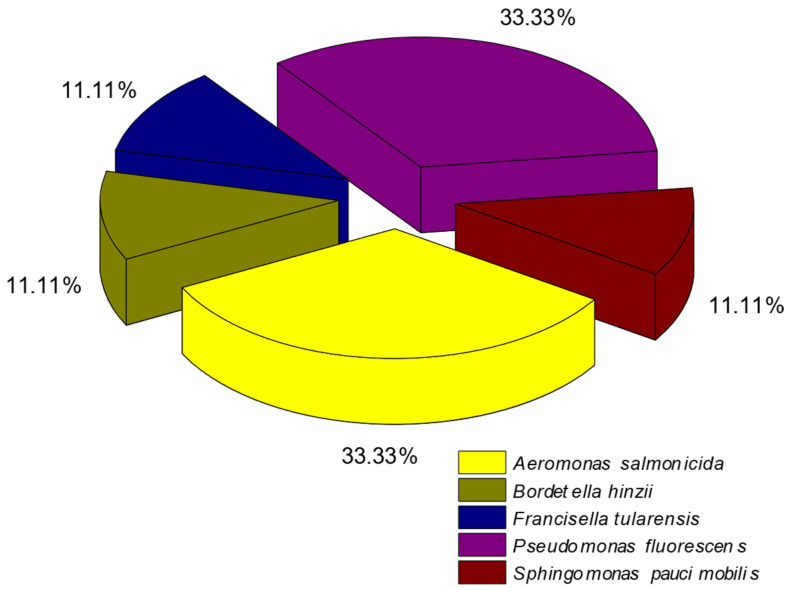
Microbial population in the case of frozen rainbow trout (*Oncorhynchus mykiss*) during the shelf life (n = 12).

Th microbial population in the frozen rainbow trout is depicted in Figure 3. The two most prevalent bacteria were *Pseudomonas fluorescens* and *Aeromonas salmonicida*, each representing 33.33% of the total bacterial population.

**Figure 4:**
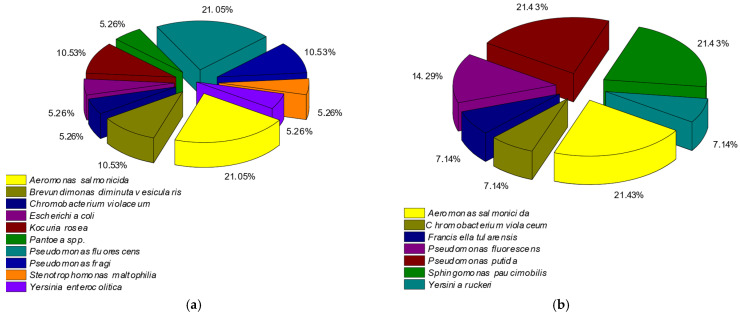
Microbial population in the case of rainbow trout (**a**) (*Oncorhynchus mykiss*) and mackerel (**b**) (*Scomber scombrus*) on flaked ice in the last day of the shelf life (n = 18).

These bacteria were followed by *Bordetella hinzii*, *Francisella tularensis*, and *Sphingomonas paucimobilis*, with 11.11% each. In the rainbow trout with one day of ice storage, we identified four species of bacteria (Figure 3). The two most prevalent bacteria were *Aeromonas salmonicida* and *Pseudomonas fluorescens*, with 33.33% each. Then came *Acinetobacter lwofii*, with 22.22%, followed by *Yersinia kristensenii*, with 11.11%. In the rainbow trout with twelve days of ice storage, we identified ten species of bacteria (Figure 4). The two most prevalent bacteria were *Aeromonas salmonicida* and *Pseudomonas fluorescens*, with 21.05% each. Then came *Pseudomonas fragi*, *Kocuria rosea*, and *Brevundimonas diminuta vesicularis*, with 10.53% each. These were followed by *Escherichia coli*, *Pantoea* spp., *Chromobacterium violaceum*, *Stenotrophomonas maltophilia*, and *Yersinia enterocolitica* at 5.26%.

In the mackerel with eleven days of ice storage, we identified seven species of bacteria (Figure 4). The three most prevalent bacteria were *Aeromonas salmonicida*, *Pseudomonas putida* and *Sphingomonas paucimobilis*, with 21.43% each. Then was *Pseudomonas fluorescens*, with 14.29%, followed by *Chromobacterium violaceum*, *Francisella tularensis*, and *Yersinia ruckeri*, with 7.14% each.

### 3.3. Pathogens Detection in Mackerel Samples

Three samples showed specific *Salmonella* spp. colonies after incubation on XLD and Rambach agar, but none of them were positive for *Salmonella* enteritidis- or *Salmonella* thyphimurium-specific sequences. In the case of *Listeria* spp., colonies developed after incubation on ALOA and Oxford agar and based on chemical confirmation, *Listeria innocua* was detected in 5.55% (two samples) and *Listeria welshimeri* in 3.33% (one sample). *Listeria monocytogenes* was not detected in any of the samples analyzed and was negative at PCR for specific sequences.

### 3.4. Histamine Production

**Figure 5:**
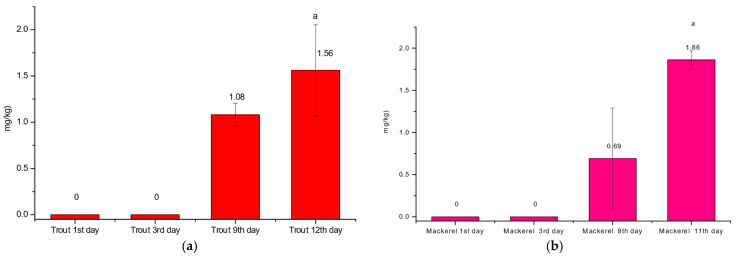
Mean histamine level in the case of rainbow trout (**a**) (*Oncorhynchus mykiss*) and mackerel (**b**) (*Scomber scombrus*) on flaked ice during the shelf life (n = 36, 12 samples/replicate). ^a^ Significant differences (*p* < 0.05) when compared with the 9th and 11/12th day.

The results regarding histamine levels in the rainbow trout on flaked ice during the shelf life are depicted in Figure 5. Histamine levels increase from the first and the third day, where no histamine was detected, to the twelfth day, with a histamine level of 1.56077 ± 0.49254 mg/kg. The results regarding histamine levels in the mackerel on flaked ice during the shelf life are depicted in Figure 5b. Histamine levels increase from the first and the third day, where no histamine was detected, to the eleventh day, with a histamine level of 1.86667 ± 0.10693 mg/kg. In both trout and mackerel samples, statistical differences were noticed between the ninth and the last day of the experiment (*p* < 0.05).

**Figure 6:**
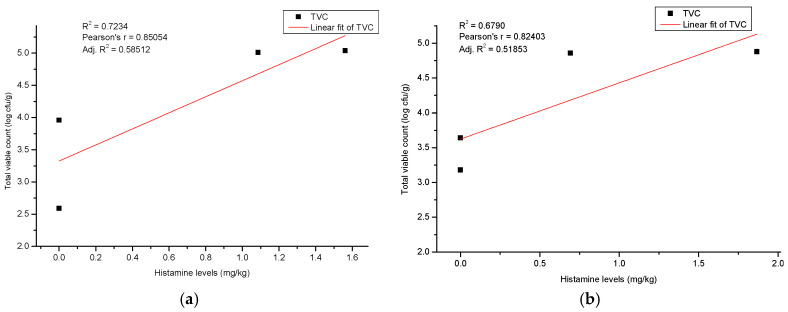
Positive correlation between total viable count and histamine values in the case of rainbow trout (**a**) (*Oncorhynchus mykiss*) and mackerel (**b**) (*Scomber scombrus*) on flaked ice during the shelf life.

The correlations between the total viable count and the histamine level in the case of the rainbow trout and mackerel on flaked ice are depicted in Figure 6a,b.

The storage timeline begins with the first day at the bottom left, showing a histamine value of 0 mg/kg and a TVC of 2.59 log cfu/g. The next point, representing the third day, is slightly above, with a histamine value of 0 mg/kg and a TVC of 3.96 log cfu/g. On the ninth day, the data point to the right shows a histamine value of 1.09 mg/kg and a TVC of 5.01 log cfu/g, and finally, the twelfth day is marked at the top right with a histamine value of 1.56 mg/kg and a TVC of 5.04 log cfu/g. A positive correlation between TVC and histamine is observed (Pearson’s r = 0.85054; Adj. R-Square = 0.58512), indicating that an increase in TVC is associated with a rise in histamine levels. Similarly, the correlation between total viable count and histamine levels in mackerel on flaked ice, shown in Figure 6b, starts with the first day at the bottom left, with a histamine value of 0 mg/kg and a TVC of 3.18 log cfu/g. The third day is represented by the next dot above, with a histamine value of 0 mg/kg and a TVC of 3.64 log cfu/g. The ninth day shows a histamine value of 0.69 mg/kg and a TVC of 4.86 log cfu/g, while the twelfth day is marked at the top right, with a histamine value of 1.87 mg/kg and a TVC of 4.88 log cfu/g. There is a positive correlation between TVC and histamine here as well (Pearson’s r = 0.82403; Adj. R-Square = 0.51853), reflecting that an increase in TVC is accompanied by a rise in histamine levels.

## 4. Discussion

Our study observed a consistent increase in microbial populations in both rainbow trout and mackerel as storage time on flaked ice progressed. This is consistent with previous findings that bacterial proliferation begins immediately after fish death, even under refrigerated conditions [34,35]. However, some species-specific differences in microbial growth dynamics and histamine accumulation were noted, likely influenced by intrinsic properties such as fat content, habitat (freshwater vs. marine), and initial microbial flora.

The total viable count (TVC) reached a maximum of 5.04 log cfu/g in rainbow trout and 4.88 log cfu/g in mackerel by the end of the storage period. While both remained under the acceptable spoilage threshold of 10^7^ cfu/g [36], the slightly higher microbial load in rainbow trout may be attributed to its leaner flesh, which tends to be more susceptible to rapid bacterial colonization due to lower levels of antimicrobial lipids compared to fatty fish like mackerel. The detection of *Enterobacteriaceae*, particularly *Escherichia coli* after twelve days of storage, suggests the fish may have been improperly handled, caught in polluted waters, or stored with contaminated ice [37,38]. On the other hand, mackerel, being a fatty marine species, may have initially had a different composition of spoilage-associated microbiota, influencing its slightly slower microbial increase despite being more prone to lipid oxidation and associated spoilage mechanisms.

Specific spoilage organisms such as *Pseudomonas* and *Aeromonas* were prevalent in both species, yet their abundance was higher in rainbow trout, particularly after day 9. These bacteria are known to dominate in freshwater environments and may have found more favorable conditions in trout due to lower competitive marine microbiota and different skin/mucus characteristics. Conversely, the presence of *Listeria innocua* and *Listeria welshimeri* exclusively in mackerel samples suggests potential differences in environmental contamination routes or handling practices specific to marine fish, possibly related to post-harvest processing or storage in marine-sourced ice. Our findings revealed that all bacteria in the sample were Gram-negative, except for *Kocuria rosea*, the only Gram-positive bacterium identified. This is consistent with studies on fish from temperate waters, where Gram-negative bacteria predominate [39,40,41]. Similar to the previous literature, we found that the dominant spoilage bacteria in ice-stored fish were *Pseudomonas* and *Aeromonas* spp. However, unlike other studies, we did not detect *Shewanella putrefaciens*, another known major spoilage bacterium [42].

We also identified bacteria typically found on fish, some of which are considered fish pathogens. These included *Aeromonas salmonicida*, *Pseudomonas* spp., *Francisella* spp., and *Yersinia* spp. *Aeromonas salmonicida*, a pathogen in fish found in both fresh and saltwater that has been associated with human diseases [42]. *Pseudomonas* spp., represented by *P. fluorescens*, *P. fragi*, and *P. putida*, is ubiquitous in soil and water and can be found in refrigerated foods. These bacteria are known to be pathogenic in both fish and humans [42,43,44,45], and a correlation has been suggested between *P. fluorescens* in the human gastrointestinal tract and Crohn’s disease [46,47]. *Francisella* spp. are fish pathogens [48,49], and *Francisella tularensis* can infect humans through various routes, including ingestion and inhalation, with a low infectious dose [50,51,52]. *Yersinia* spp., including *Y. ruckeri*, *Y. enterocolitica*, and *Y. kristensenii*, are also of concern as they may cause fish diseases and pose a zoonotic risk [53,54,55,56,57].

Other bacteria identified in the study are not commonly found in living fish but are typically environmental, including *Sphingomonas paucimobilis*, *Brevundimonas diminuta*, *Chromobacterium violaceum*, *Acinetobacter lwoffii*, *Kocuria rosea*, *Pantoea* spp., and *Steno-trophomonas maltophilia*. These bacteria are mostly opportunistic pathogens, often causing infections in immunocompromised individuals [47,58,59,60]. For example, *S. paucimobilis* is a low-virulence bacterium mainly associated with nosocomial infections [59], while *Brevundimonas* species have been isolated from various environments and are opportunistic pathogens [61,62]. *Acinetobacter* species, commonly found in water and soil, are part of the normal flora but also cause nosocomial infections [63]. *Kocuria rosea* has been linked to human infections in recent years [64,65]. *Pantoea* spp. are opportunistic pathogens associated with nosocomial infections [66], while *Stenotrophomonas maltophilia* is another opportunistic pathogen that has been linked to nosocomial infections [67].

In contrast to our findings, Jamali et al. (2015) [68] reported higher contamination levels, with a prevalence of *Listeria innocua* at 35.3%, while Malakar et al. (2020) [69] found lower contamination (16.21%) in fish and meat samples. Though *Listeria innocua* is non-pathogenic to humans, it has been implicated in cases of listeriosis, highlighting its zoonotic potential [70]. The presence of *E. coli*, *Listeria innocua*, and *Listeria welshimeri* in some mackerel samples suggests potential contamination during processing or storage, with variations in microbial load due to differences in handling and environmental conditions.

Histamine production increased in both fish species over time but remained well below the maximum limit (<200 mg/kg) [71]. Notably, mackerel showed a slightly higher peak histamine level (1.87 mg/kg) compared to rainbow trout (1.56 mg/kg), possibly due to its higher histidine content, a known precursor to histamine. The fatty nature of mackerel may also support certain histamine-producing bacterial species better than lean fish. A strong positive correlation between TVC and histamine was observed in both species but more markedly in trout (r = 0.85 vs. r = 0.82), suggesting potential differences in the dominant histamine-producing microbiota. While both species showed microbial and chemical spoilage trends aligned with the general storage behavior of chilled fish, these nuanced differences underscore the importance of tailoring post-harvest handling and storage strategies according to fish species. The lean and freshwater characteristics of rainbow trout may predispose it to faster microbial proliferation, while the fatty and marine nature of mackerel influences both spoilage microbiota and histamine dynamics in distinct ways.

According to the literature, toxic levels of histamine require a bacterial load of 10^8^ cfu/g [72,73], which is higher than the maximum total viable count observed in our samples (5.04 log cfu/g for rainbow trout and 4.88 log cfu/g for mackerel). Our findings align with a study by Dawood et al. (1988) [74], which showed that rainbow trout stored at 0 °C for twelve days after temperature abuse had histamine levels below those necessary for intoxication. Interestingly, a positive correlation between total viable count and histamine was not found in the literature. For example, Kim et al. (1999) [75] did not observe histamine in fish stored in ice for up to 18 days, and other studies suggest that establishing a correlation between viable count and histamine is difficult due to the involvement of various factors, such as the initial histamine-producing bacteria and the diverse species of bacteria responsible for histamine production and degradation [76].

## 5. Conclusions

This study demonstrated that both rainbow trout (lean, freshwater) and Atlantic mackerel (fatty, marine) stored on flaked ice experienced progressive microbial growth and increased histamine formation over time. Despite differences in fat content and origin, both species remained within acceptable microbiological and chemical safety limits throughout the studied shelf life. However, rainbow trout showed slightly higher microbial loads, while mackerel had marginally higher histamine levels by the end of storage.

These differences are likely rooted in the distinct biological and environmental variations and highlight the importance of species-specific handling protocols and storage conditions to maintain fish safety and quality.

Based on microbial and histamine trends, both species should ideally be consumed before the ninth day of storage on flaked ice. Implementing good hygiene practices (GHPs) and hazard analysis and critical control points (HACCPs) tailored to the specific risks associated with each species remains essential to mitigate contamination and spoilage risks. Further comprehensive studies are necessary to evaluate the safety of fresh fish stored on flaked ice that is intended for public consumption.

## Data Availability

Data are unavailable due to privacy or ethical restrictions.

## References

[1] Abisha R., Krishnani K.K., Sukhdhane K., Verma A.K., Brahmane M., Chadha N.K. (2022). Sustainable Development of Climate-Resilient Aquaculture and Culture-Based Fisheries Through Adaptation of Abiotic Stresses: A Review. J. Water Clim. Change.

[2] Khalili Tilami S., Sampels S. (2017). Nutritional Value of Fish: Lipids, Proteins, Vitamins, and Minerals. Rev. Fish. Sci. Aquacult..

[3] Pila M., Stanciuc N., Stanciu S. (2023). Fisheries and Aquaculture in Romania: A Global Outlook on Sustainable Development. Sci. Pap. Ser. Manag. Econ. Eng. Agric. Rural Dev..

[4] Tahiluddin A., Maribao I., Amlani M., Sarri J. (2022). A Review on Spoilage Microorganisms in Fresh and Processed Aquatic Food Products. Food Bull..

[5] Egerton S., Culloty S., Whooley J., Stanton C., Ross R.P. (2018). The Gut Microbiota of Marine Fish. Front. Microbiol..

[6] Powers N.C., Wallgren H.R., Marbach S., Turner J.W. (2020). Relationship between Rainfall, Fecal Pollution, Antimicrobial Resistance, and Microbial Diversity in an Urbanized Subtropical Bay. Appl. Environ. Microbiol..

[7] Riedel T.E., Thulsiraj V., Zimmer-Faust A.G., Dagit R., Krug J., Hanley K.T., Adamek K., Ebentier D.L., Torres R., Cobian U. (2015). Long-Term Monitoring of Molecular Markers Can Distinguish Different Seasonal Patterns of Fecal Indicating Bacteria Sources. Water Res..

[8] Bell G.R., Hoskins G.E., Hodgkiss W. (1971). Aspects of the Characterization, Identification, and Ecology of the Bacterial Flora Associated with the Surface of Stream-Incubating Pacific Salmon (*Oncorhynchus*) Eggs. J. Fish. Res. Bd. Can..

[9] Bolnick D.I., Snowberg L.K., Hirsch P.E., Lauber C.L., Knight R., Caporaso J.G., Svanbäck R. (2014). Individuals’ Diet Diversity Influences Gut Microbial Diversity in Two Freshwater Fish (Threespine Stickleback and Eurasian Perch). Ecol. Lett..

[10] Dehler C.E., Secombes C.J., Martin S.A.M. (2017). Environmental and Physiological Factors Shape the Gut Microbiota of Atlantic Salmon Parr (*Salmo salar* L.). Aquaculture.

[11] Blanch A., Alsina M., Simon M., Jofre J. (1997). Determination of Bacteria Associated with Rearing Turbot (*Scophthalmus maximus*) Larvae. J. Appl. Microbiol..

[12] Burr G., Delbert G., Steven R. (2005). Microbial Ecology of the Gastrointestinal Tract of Fish and the Potential Application of Prebiotics and Probiotics in Finfish Aquaculture. J. World Aquac. Soc..

[13] Karthiayani T., Mahadeva Iyer K. (1967). Quantitative and Qualitative Studies on the Bacterial Flora of Fresh Sardines. Fish. Technol..

[14] Larsen A.M. (2014). Studies on the Microbiota of Fishes and the Factors Influencing Their Composition. Ph.D. Thesis.

[15] Ringø E., Sperstad S., Myklebust R., Refstie S., Krogdahl Å. (2006). Characterisation of the Microbiota Associated with Intestine of Atlantic Cod (*Gadus morhua* L.). Aquaculture.

[16] Talwar C., Nagar S., Lal R., Negi R.K. (2018). Fish Gut Microbiome: Current Approaches and Future Perspectives. Indian J. Microbiol..

[17] Visciano P., Schirone M., Tofalo R., Suzzi G. (2014). Histamine Poisoning and Control Measures in Fish and Fishery Products. Front. Microbiol..

[18] Giyatmi, Irianto H.E., Kim S.-K., Toldrá F. (2017). Chapter Ten-Enzymes in Fermented Fish. Marine Enzymes Biotechnology: Production and Industrial Applications, Part III-Application of Marine Enzymes.

[19] Austin B. (2006). The Bacterial Microflora of Fish, Revised. Sci. World J..

[20] Qiu Y., Zhou Y., Chang Y., Liang X., Zhang H., Lin X., Qing K., Zhou X., Luo Z. (2022). The Effects of Ventilation, Humidity, and Temperature on Bacterial Growth and Bacterial Genera Distribution. Int. J. Environ. Res. Public Health.

[21] Bedane T.D., Agga G.E., Gutema F.D. (2022). Hygienic Assessment of Fish Handling Practices Along Production and Supply Chain and Its Public Health Implications in Central Oromia, Ethiopia. Sci. Rep..

[22] Derome N., Gauthier J., Boutin S., Llewellyn M., Hurst C. (2016). Bacterial Opportunistic Pathogens of Fish. The Rasputin Effect: When Commensals and Symbionts Become Parasitic.

[23] Novoslavskij A., Terentjeva M., Eizenberga I., Valciņa O., Bartkevičs V., Bērziņš A. (2016). Major Foodborne Pathogens in Fish and Fish Products: A Review. Ann. Microbiol..

[24] Da Costa A.R., da Costa M.M., de Carvalho Azevedo V.A., de Padua Pereira U. (2023). Fish Pathogens: Infection and Biological Control. Fishes.

[25] Irshath A.A., Rajan A.P., Vimal S., Prabhakaran V.S., Ganesan R. (2023). Bacterial Pathogenesis in Various Fish Diseases: Recent Advances and Specific Challenges in Vaccine Development. Vaccines.

[26] Su Y., Gao R., Huang F., Liang B., Guo J., Fan L., Wang A., Gao S.-H. (2024). Occurrence, Transmission, and Risks Assessment of Pathogens in Aquatic Environments Accessible to Humans. J. Environ. Manag..

[27] (2017). Microbiology of the Food Chain—Horizontal Method for the Detection, Enumeration and Serotyping of *Salmonella*. Part 1: Detection of *Salmonella* spp..

[28] (2017). Microbiology of the Food Chain—Horizontal Method for the Detection and Enumeration of *Listeria monocytogenes* and of *Listeria* spp..

[29] AOAC (1998). Determination of Histamine by Liquid-Chromatographic Method, Following the EC Regulation No. 2073/2005/EC with Its Subsequent Amendments.

[30] Lerke P.A., Werner S.B., Taylor S.L., Guthertz L.S. (1978). Scombroid Poisoning: Report of an Outbreak. West. J. Med..

[31] Frank H.A., Yoshinaga D.H., Nip W.K. (1981). Histamine Formation and Honeycombing During Decomposition of Skipjack Tuna (*Katsuwonus pelamis*), at Elevated Temperatures. Mar. Fish. Rev..

[32] Mihaiu L., Lapusan A., Tanasuica R., Sobolu R., Mihaiu R., Oniga O., Mihaiu M. (2014). First Study of *Salmonella* in Meat in Romania. J. Infect. Dev. Ctries..

[33] Duma M.N., Ciupescu L.M., Dan S.D., Crisan-Reget O.L., Tabaran A. (2024). Virulence and Antimicrobial Resistance of *Listeria monocytogenes* Isolated from Ready-to-Eat Food Products in Romania. Microorganisms.

[34] Gram L. Spoilage of Three Senegalese Fish Species Stored in Ice and at Ambient Temperature. Proceedings of the SEAFOOD 2000.

[35] Gram L., Wedell-Neergaard C., Huss H.H. (1990). The Bacteriology of Fresh and Spoiling Lake Victorian Nile Perch (*Lates niloticus*). Int. J. Food Microbiol..

[36] ICMSF (International Commission of Microbiological Specification for Food) (1986). Microorganisms in Food 2. Sampling for Microbiological Analysis: Principles and Specific Applications.

[37] Andrews W.H., Hammack T.S. (2001). BAM Chapter 1: Food Sampling/Preparation of Sample Homogenate. United States Food and Drug Administration (US FDA) Bacteriological Analytical Manual.

[38] Sanjee S.A., Karim M.E. (2016). Microbiological Quality Assessment of Frozen Fish and Fish Processing Materials from Bangladesh. Int. J. Food Sci..

[39] Renata A.C. (2013). *Escherichia coli* in Seafood: A Brief Overview. Adv. Biosci. Biotechnol..

[40] Kuley E., Durmus M., Balikci E., Ucar Y., Regenstein J.M., Ozogul F. (2017). Fish Spoilage Bacterial Growth and Their Biogenic Amine Accumulation: Inhibitory Effects of Olive By-Products. Int. J. Food Prop..

[41] Jalal K.C.A., Akbar J.B., Nurul Lyana M.S., Faizul H.N., Isma Yanti M.N., Irwandi J., Bulbul M. (2017). Comparative Study on Spoilage and Pathogenic Bacteria in Selected Commercial Marine and Freshwater Fishes. Int. Food Res. J..

[42] Altinok I., Kayis S., Capkin E. (2006). Pseudomonas putida Infection in Rainbow Trout. Aquaculture.

[43] Lamy B., Kodjo A., Laurent F. (2009). Prospective Nationwide Study of *Aeromonas* Infections in France. J. Clin. Microbiol..

[44] Bhattacharya D., Dey S., Kadam S., Kalal S., Jali S., Koley H., Sinha R., Nag D., Kholkute S.D., Roy S. (2015). Isolation of NMD-1-Producing Multidrug-Resistant *Pseudomonas putida* from a Paediatric Case of Acute Gastroenteritis, India. New Microbes New Infect..

[45] Khan F.Y., Abukamar M., Anand D. (2017). Nosocomial *Pseudomonas putida* Meningitis: A Case Report and Literature Review. Oman Med. J..

[46] Gershman M.D., Kennedy D.J., Noble-Wang J., Kim C. (2008). Multistate Outbreak of *Pseudomonas fluorescens* Bloodstream Infection after Exposure to Contaminated Heparinized Saline Flush Prepared by a Compounding Pharmacy. Clin. Infect. Dis..

[47] Hossain Z. (2014). Bacteria: *Pseudomonas*. Encycl. Food Saf..

[48] Hsieh C.Y., Tung M.C., Tu C., Chang C.D., Tsai S.S. (2006). Enzootics of Visceral Granulomas Associated with Francisella-Like Organism Infection in Tilapia (*Oreochromis* spp.). Aquaculture.

[49] Olsen A.B., Mikalsen J., Rode M., Alfjorden A., Hoel E., Straum-Lie K., Haldorsen R., Colquhoun D.J. (2006). A Novel Systemic Granulomatous Inflammatory Disease in Farmed Atlantic Cod, *Gadus morhua* L., Associated with a Bacterium Belonging to the Genus *Francisella*. J. Fish Dis..

[50] Quinn P.J., Markey B.K., Carter M.E., Donnelly W.J., Leonard F.C. (2002). Veterinary Microbiology and Microbial Disease.

[51] Pechous R.D., McCarthy T.R., Zahrt T.C. (2009). Working Toward the Future: Insights into *Francisella tularensis* Pathogenesis and Vaccine Development. Microbiol. Mol. Biol. Rev..

[52] Johansson A., Forsman M., Liu D. (2018). Chapter 31: Francisella. Handbook of Foodborne Diseases. Food Microbiology.

[53] De Keukeleire S., De Bel A., Jansen Y., Janssens M., Wauters G., Piérard D. (2014). *Yersinia ruckeri*, an Unusual Microorganism Isolated from a Human Wound Infection. New Microbes New Infect..

[54] Kumar G., Menanteau-Ledouble S., Saleh M., El-Matbouli M. (2015). *Yersinia ruckeri*, the Causative Agent of Enteric Redmouth Disease in Fish. Vet. Res..

[55] EFSA (European Food Safety Agency) (2007). The Community Summary Report on Trends and Sources of Zoonoses, Zoonotic Agents, Antimicrobial Resistance, and Foodborne Outbreaks in the European Union in 2006. EFSA J..

[56] Shanmugapriya S., Senthilmurugan T., Thayumanavan T. (2014). Genetic Diversity Among *Yersinia enterocolitica* Isolated from Chicken and Fish in and Around Coimbatore City, India. Iran. J. Public Health.

[57] Wang L. (2016). Yersinia enterocolitica as a Cause of Septicemia in Crucian Carp (*Carassius carassius*). Iran. J. Fish. Sci..

[58] Ryan M.P., Adley C.C. (2010). *Sphingomonas paucimobilis*: A Persistent Gram-Negative Nosocomial Infectious Organism. J. Hosp. Infect..

[59] Steinberg J.P., Burd E.M. (2015). Chapter 238: Other Gram-Negative and Gram-Variable Bacilli. Mandell, Douglas, and Bennett’s Principles and Practice of Infectious Diseases.

[60] Ryan M.P., Pembroke J.T. (2018). *Brevundimonas* spp: Emerging global opportunistic pathogens. Virulence.

[61] Li Y., Kawamura Y., Fujiwara N., Naka T., Liu H., Huang X., Kobayashi K., Ezaki T. (2004). Sphingomonas yabuuchiae sp. nov. and Brevundimonas nasdae sp. nov., isolated from the Russian space laboratory Mir. Int. J. Syst. Evol. Microbiol..

[62] Kang S.J., Choi N.-S., Choi J.H., Lee J.-S., Yoon J.-H., Song J.J. (2009). *Brevundimonas naejangsanensis* sp. nov., a proteolytic bacterium isolated from soil, and reclassification of *Mycoplana bullata* into the genus *Brevundimonas* as *Brevundimonas bullata* comb. nov. Int. J. Syst. Evol. Microbiol..

[63] Rathinavelu S., Zavros Y., Merchant J.L. (2003). Acinetobacter lwoffii infection and gastritis. Microbes Infect..

[64] Altuntas F., Yildiz O., Eser B., Gundogan K., Sumerkan B., Cetin M. (2004). Catheter-related bacteremia due to *Kocuria rosea* in a patient undergoing peripheral blood stem cell transplantation. BMC Infect. Dis..

[65] Ma E.S., Wong C.L., Lai K.T., Chan E.C., Yam W.C., Chan A.C. (2005). *Kocuria kristinae* infection associated with acute cholecystitis. BMC Infect. Dis..

[66] Cunningham D.J., Leber A. (2018). 140-Enterobacter, Cronobacter, and Pantoea Species. Principles and Practice of Pediatric Infectious Diseases.

[67] Denton M., Kerr K.G. (1998). Microbiological and Clinical Aspects of Infection Associated with *Stenotrophomonas maltophilia*. Clin. Microbiol. Rev..

[68] Jamali H., Paydar M., Ismail S., Looi C.Y., Wong W.F., Radmehr B., Abedini A. (2015). Prevalence, antimicrobial susceptibility and virulotyping of *Listeria* species and *Listeria monocytogenes* isolated from open-air fish markets. BMC Microbiol..

[69] Malakar D., Borah P., Das L., Mathipi V., Sailo C.V., Dutta R., Deka N.K., Kumar N.S. (2020). Prevalence and Virulent Gene Profiling of *Listeria monocytogenes* from Fish and Meat Samples from Aizawl, Mizoram. J. Pure Appl. Microbiol..

[70] Ramadan H., Al-Ashmawy M., Soliman A.M., Elbediwi M., Sabeq I., Yousef M., Algammal A.M., Hiott L.M., Berrang M.E., Frye J.G. (2023). Whole-genome sequencing of *Listeria innocua* recovered from retail milk and dairy products in Egypt. Front. Microbiol..

[71] European Commission (2005). Commission Regulation (EC) No 2073/2005 of 15 November 2005 on microbiological criteria for foodstuffs. Off. J. Eur. Union..

[72] Fletcher G.C., Summers G., Winchester R.V., Wong R.J. (1995). Histamine and Histidine in New Zealand Marine Fish and Shellfish Species, Particularly Kahawai (*Arripis trutta*). J. Aquat. Food Prod. Technol..

[73] Food and Agriculture Organization (FAO) (2004). Application of Risk Assessment in the Fish Industry.

[74] Dawood A.A., Karkalas J., Roy R.N., Williams C.S. (1988). The Occurrence of Non-volatile Amines in Chilled-stored Rainbow Trout (*Salmo irideus*). Food Chem..

[75] Kim S.H., An H., Price R.J. (1999). Histamine Formation and Bacterial Spoilage of Albacore Harvest off the U.S Northwest Coast. J. Food Sci..

[76] Lehane L., Olley J. (2000). Histamine Fish Poisoning Revisited. Int. J. Food Microbiol..

